# Photosynthetic microorganisms effectively contribute to bryophyte CO_2_ fixation in boreal and tropical regions

**DOI:** 10.1038/s43705-022-00149-w

**Published:** 2022-07-28

**Authors:** Vincent E. J. Jassey, Samuel Hamard, Cécile Lepère, Régis Céréghino, Bruno Corbara, Martin Küttim, Joséphine Leflaive, Céline Leroy, Jean-François Carrias

**Affiliations:** 1 Laboratoire Ecologie Fonctionnelle et Environnement (LEFE), Université Toulouse 3—Paul Sabatier (UT3), CNRS, 31062 Toulouse, France; 2 Laboratoire Microorganismes, Génome Et Environnement (LMGE), Université Clermont Auvergne, CNRS, Clermont-Ferrand, France; 3 Institute of Ecology, School of Natural Sciences and Health, Tallinn University, Uus-Sadama 5, 10120 Tallinn, Estonia; 4 AMAP, Univ Montpellier, CIRAD, CNRS, INRAE, IRD, Montpellier, France; 5 ECOFOG, AgroParisTech, CIRAD, CNRS, INRAE, Université de Guyane, Université des Antilles, Campus Agronomique, Kourou, France

**Keywords:** Community ecology, Physiology, Microbial ecology

## Abstract

Photosynthetic microbes are omnipresent in land and water. While they critically influence primary productivity in aquatic systems, their importance in terrestrial ecosystems remains largely overlooked. In terrestrial systems, photoautotrophs occur in a variety of habitats, such as sub-surface soils, exposed rocks, and bryophytes. Here, we study photosynthetic microbial communities associated with bryophytes from a boreal peatland and a tropical rainforest. We interrogate their contribution to bryophyte C uptake and identify the main drivers of that contribution. We found that photosynthetic microbes take up twice more C in the boreal peatland (~4.4 mg CO_2_.h^−1^.m^−2^) than in the tropical rainforest (~2.4 mg CO_2_.h^−1^.m^−2^), which corresponded to an average contribution of 4% and 2% of the bryophyte C uptake, respectively. Our findings revealed that such patterns were driven by the proportion of photosynthetic protists in the moss microbiomes. Low moss water content and light conditions were not favourable to the development of photosynthetic protists in the tropical rainforest, which indirectly reduced the overall photosynthetic microbial C uptake. Our investigations clearly show that photosynthetic microbes associated with bryophyte effectively contribute to moss C uptake despite species turnover. Terrestrial photosynthetic microbes clearly have the capacity to take up atmospheric C in bryophytes living under various environmental conditions, and therefore potentially support rates of ecosystem-level net C exchanges with the atmosphere.

## Introduction

Over the last decade, environmental DNA studies revealed that terrestrial systems harbour diverse microbial communities [1–4], with significant implications in biogeochemical cycles [5]. Most microorganisms use organic carbon (C) derived from vegetation or predation as energy and carbon sources [6]. These organotrophs are highly involved in terrestrial C releases such as CO_2_ and CH_4_ respiration fluxes at the global scale [7]. However, many photosynthetic terrestrial bacteria and protists use inorganic CO_2_ in addition to light as an energy source [6, 8, 9]. While these photosynthetic microbes have key ecological and biogeochemical roles [10–13], they are thought to make a minor contribution to terrestrial primary productivity, compared to plants (but see refs. [14, 15]).

Emerging evidence has shown that photosynthetic microbes occur in a variety of terrestrial habitats, including sub-surface soils [15–17], exposed rocks [18], and bryophytes [19]. They encompass myriads of life forms and styles, with Cyanobacteria and Chlorophyta being the most commonly reported phyla in DNA-based global diversity surveys [2, 20]. Most of our knowledge about terrestrial photosynthetic microbes comes from drylands [21, 22], such as hot and cold deserts, where they build biological crusts on soil surface [17]. Generally, these crusts are dominated by one large group of photosynthetic organisms, such as Cyanobacteria in high pH environments and Chlorophyta in more neutral to acidic environments [23], although other taxonomic groups such as Bacillariophyta, Eustigmatophyceae, and Xanthophyceae can also be commonly found but in lower proportions [2, 23]. More mesic ecosystems have been less studied than arid systems despite regular microscopic observations and DNA hits showing that photosynthetic microbes occur in other terrestrial biomes [2]. More particularly, high abundances of photosynthetic microbes have been found in peatlands [9, 13, 24] and tropical rainforests [25], where they regularly colonize bryophytes.

Bryophytes play a central role in many mesic ecosystems, as they form a zone of nutrient accumulation and transformation [26]. Widespread and abundant in many terrestrial ecosystems, the morphological and ecophysiological attributes of bryophytes allow them to grow in habitats that most vascular plants cannot colonize such as water, exposed rocks and soil, tree trunks, and leaves. Bryophytes are thus a conspicuous component of the understory vegetation of many forest ecosystems [27, 28], and even the dominant vegetation of wetlands such as peatlands [29]. Like all plants, bryophytes associate with microbes. These microbial communities are involved in the development, growth, and health—in other words, all functions—of bryophytes [26, 30–32]. The individual phenotype of bryophytes is indeed the result of complex interactions between the combined expression of the host and associated microbiomes, which together form the so-called bryosphere [26].

Bryophyte-associated microbiomes are involved in multiple ecosystem-level processes such as N_2_ fixation [32] and methane oxidation [33]. For these reasons, there has long been an interest in understanding what bryophyte-associated microbes do, and what is their contribution to ecosystem processes. Through atmospheric CO_2_ uptake and cycling of nutrients, photosynthetic microbes serve as elemental sinks in the bryosphere by providing nutrients for plants [32, 34], and food for animals [11] and other microorganisms [10, 35]. Moreover, photosynthetic microbes are likely to modulate bryosphere C flux exchange with the atmosphere. They can support bryosphere C uptake through their photosynthetic CO_2_ uptake [9] and affect soil CO_2_ effluxes by stimulating bacterial respiration and biomass through their exudates [13], although the later strongly depends on the amount of C they initially fixed. Therefore, questions arise about the carbon fixation rates of photosynthetic microbes in bryophytes.

Here, we explore to what extent the photosynthetic microbial C assimilation rate contributes to the whole bryosphere C fixation and identify the main microbial drivers (diversity, abundance, photosynthetic capacity) of that contribution. To this end, we studied two contrasting types of bryosphere, i.e. *Sphagnum* mosses in a boreal peatland and feather mosses in a tropical rainforest. We assessed how the specific features of the bryosphere (e.g. taxonomy, incoming light intensity, moisture content, and global climate) relate to compositional differences in 16 S and 18 S rDNA-derived photosynthetic microbial communities (photosynthetic bacteria and protists) and how the turnover in 16 S and 18 S communities affects the total photosynthetic microbial abundance and C uptake. We further estimated the contribution of photosynthetic microbial C uptake to the whole bryosphere C fixation by conducting CO_2_ gas exchange measurements. We acknowledge that comparing the bryosphere of a tropical rainforest to that of a boreal peatland may seem *a priori* counterintuitive because of differences in environmental matrices and bryosphere ecology. Yet this comparison not only provides basic information on the diversity and community structure of overlooked terrestrial microorganisms, but it does so by analysing diversity-function relationships and metabolic processes relative to microbes within bryospheres, making our cross-ecosystem study relevant for improving our understanding of the role of photosynthetic microbes to C cycling across regions of the world. We hypothesize that current bryosphere properties reflect variations in photosynthetic microbial communities, which in turn shape their C fixation rates and contribution to total bryosphere C fixation. For example, we know that some cyanobacteria are tolerant to environmental conditions such as daily hydration/dehydration cycles, low irradiance, and high temperatures [36, 37]—in other words, the climatic conditions usually found in tropical rainforests. We, therefore, expect cyanobacteria to dominate in the tropical rainforest, which could translate into contrasting microbial C fixation rates as cyanobacteria use light less efficiently than most photosynthetic micro-eukaryotes.

## Materials and methods

### Sites description and sampling

This study was carried out in French Guiana (tropical rainforest) and Estonia (boreal peatland) in late March and late September 2019, respectively. Our study site in French Guiana was a primary forest near the Petit-Saut Dam, Sinnamary (5°03043”N, 53°0246”W; elevation <80 m a.s.l). French Guiana is located on the north-eastern coast of South America. The climate is tropical moist with 2451 mm of annual precipitation, with little seasonal variation in air temperature (mean annual temperature = 26 °C, monthly average = 20.5–33.5 °C), and relative humidity (>70%). Our study area was characterized by a high abundance of epiphytic bryophytes, which germinate and grow on tree trunks (Table 1). To explore the diversity of bryophyte-associated photosynthetic microbes within the forest, we conducted our survey on 25 randomly selected tree trunks dominated and/or co-dominated by the bryophytes *Lejeunea cavifolia, Leucolejeunea clypeata* and *Syyrhopodon* sp. (Table 1). In late March 2019, we sampled the bryophytes (living part only) on each tree at human height (~1.7 m height) for analyses of photosynthetic microbial diversity, abundance, and photosynthetic rates as well as the bryosphere C fixation rates (see below for details).

**Table 1:** Local and environmental variables characterising the different bryospheres habitats.

		Habitat	Dominant species	Light intensity (LUX)	Bryophyte water content g H2O2/g dw ± SD	MAT (°C)	MAP (mm)
	Tropical rainforest	Tree trunks (*n* = 25)	*Lejeunea cavifolia*, *Leucolejeunea clypeata*, *Syyrhopodon* sp.	5015 ± 368	1.4 ± 1.5	26.0	2451
	Boreal peatland	Lawn (*n* = 3)	*S. medium*, *S. balticum*	23647 ± 4353	19.3 ± 3.1	5.1	710
		Hummock (*n* = 3)	*S. fuscum*	20368 ± 2340	10.6 ± 1.4		
		Wooded hummock (*n* = 3)	*S. fuscum*	14342 ± 7098	11.4 ± 1.6		
		Forest ditch (*n* = 3)	*S. riparium*	11705 ± 1469	37.4 ± 9.7		

*MAT* mean annual temperature, *MAP* mean annual precipitation.

Our boreal site was situated in central Estonia at Männikajärve raised bog (58°52’30 N, 26°15’04 E, 78 m a.s.l.), where the mean long-term (1962–2019) annual temperature and precipitation are 5.1 °C and 710 mm, respectively (Estonian Weather Service). The site is characterized by vegetation dominated by bryophytes from the *Sphagnum* genus and patchy vascular plant layer [38], with the exception of the edges of the site where *Pinus sylvestris*, *Picea abies* and *Betula pendula* were more abundant (Table 1). To take into account the diversity of bryophyte-associated photosynthetic microbes within the peatland, we sampled *Sphagnum* bryophytes across four microhabitats (3 plots per microhabitat; 12 plots in total), i.e., lawns, hummocks, wooded hummocks, and forest ditches, that differ in terms of *Sphagnum* species and light intensity at the ground surface (see Table 1). In late September 2019, we sampled *Sphagnum* shoots (living part only) in each plot for analysis of *Sphagnum* C fixation rates as well as photosynthetic microbial diversity, abundance, and photosynthetic rates.

At each site, all samples have been collected on the same day, and C fluxes were measured when the sun was at its highest (between 11 and 13 a.m). Sampling adequately captured the mean annual microclimate conditions of the bryosphere in each site, especially in terms of precipitation (Fig. S1). To characterize bryosphere microclimatic conditions in each plot, we measured the bryophyte water content (WC) by collecting 12 cm^2^ of fresh bryophyte on the day of sampling, weighting it fresh, and drying it for two days at 60 °C. Bryophyte WC was expressed in gram of H_2_O per dry mass of bryophyte (g H_2_O/g^−1^ dm). We also measured light intensity using a lux meter in the peatland (LI-COR Li-189, USA) and a PAR sensor in the tropical forest (Table 1). One measure has been made in the centre of each plot while sampling.

### Photosynthetic diversity, community structure, and abundance

We assessed the photosynthetic microbial diversity and community structure by means of high-throughput sequencing. 16 S and 18 S rRNA gene markers were used for photosynthetic bacteria and protists, respectively. We also included mixotrophic ciliates and testate amoebae in our analyses as they also perform photosynthesis. All details related to DNA extraction, amplification, and bioinformatic pipeline are given in Supplementary Method and Table S1. To assess photosynthetic microbial abundance, we sampled approximately 3 grams of homogenized bryophyte material (living moss only) in each plot, fixed them in 10 ml of formaldehyde (4% final concentration), and stored them at 4 °C in the dark until microscopic analyses. Microorganisms were extracted from bryophytes and enumerated using inverted microscopy (Zeiss Axiovert 200 M, Carl Zeiss company, Oberkochen, Germany) by means of the plankton chamber approach and following standardized protocols [39]. Because of the high abundance of cyanobacteria in tropical rainforest samples, we further combined inverted microscopy with epifluorescence to better distinguish the autofluorescence of chlorophyll a (blue light excitation, 450–490 nm) and phycoerythrin (green light excitation, 520–560 nm) to differentiate pigmented protists from cyanobacteria in these samples. Photosynthetic microbial abundance data were expressed as the number of photosynthetic microbe per gram of bryophyte dry weight (ind.g^−1^ DW).

### Microbial chlorophyll-*a* concentration, photosynthesis efficiency, and CO_2_ fixation rate

Microbial photosynthetic rates were quantified and calculated using pulse amplitude modulation (PAM) fluorometry, as described in [9]. Briefly, approximately 4 grams of homogenized living bryophyte material were sampled in each plot, immersed in 25 ml demineralized water, and gently shaken intermittently for 1 min by hand. Then, the solution was passed through a 100 *µ*m nylon mesh to remove any bryophyte material and filtered through a GF/F Whatman^®^ filter. The remaining bryophyte material was dried at 60 °C for 48 h and weighed. GF/F Whatman^®^ filters containing the microbial communities were dark-adapted for 30 min and then exposed to increasing light to measure the light curve of the quantum yield of photosystem II (ΦPSII) using a Phyto-PAM (Walz, Effeltrich, Germany). The chlorophyll-*a* content of microbial communities was assessed by HPLC following [9]. Microbial chlorophyll-*a* (Chla) was expressed in ng per gram of dry bryophyte. It was then divided by the total abundance of photosynthetic microbes to estimate the chlorophyll-*a* content per microbial cell (expressed in ng.cell^−1^). We estimated the microbial photosynthetic rate in each plot by calculating the photosynthetic electron transport rate per microbial cell (ETR, mol e- cell^−1^ s^−1^) as detailed in [40], and assuming a maximum fixation of 0.25 mol CO_2_ per mol of electron [40].

### Bryophyte C fixation rates

In each plot, we measured the bryophyte photosynthetic rates (CO_2_ assimilation rate) *in situ* as described in ref. [41]. The net CO_2_ assimilation was measured with an open‐path infrared gas analyser (IRGA) system connected to a 2.5 cm^2^ PLC‐5 chamber (TARGAS-1; PP‐Systems) under field light and temperature conditions, and by making sure the vapor pressure deficit was <1 kPa during measurements. In each plot, we collected enough moss to fill the entire area of the PLC-5 chamber (approximately 2 grams of fresh mass). Immediately after measurements, the moss samples have been dried at 60 °C for two days and their weight was measured to express the photosynthetic rates per dry mass of bryophyte. Finally, we converted the photosynthetic rate of the bryosphere from *µ*mol.s^−1.^g^−1^ CO_2_ to mg CO_2_ h^−1^ m^−2^, using the WC content expressed per surface area, thus allowing comparisons with microbial C fixation rates. To estimate the relative contribution (in percent) of photosynthetic microbes to the bryosphere CO_2_ fixation, we divided the microbial CO_2_ fixation rate by the bryosphere CO_2_ fixation rate (both expressed in mg CO_2_ h^−1^ m^−2^) and multiplied it by 100.

### Statistical analyses

Merging microbial sequence data from disparate studies can generate some bias in data analyses, resulting from methodological issues regarding primer choice, sequencing depth, and PCR bias [42–44]. DNA sequencing of the rainforest and peatland samples were initially conducted to stand as separate studies, and as such, show some methodological discrepancies (i.e. different primers pair for 18 S). Although recent findings showed that disparate amplicon sequence data can be combined at the taxonomy level to assess macroecological patterns in microbial community structure [45], we took into consideration potential bias that could have emerged from this merging in our analyses using random forests models (see Supplementary Method). To test whether photosynthetic prokaryotes and protists communities were specific to the rainforest or the peatland, we performed a permutational multivariate analysis of variance coupled with non-metric multidimensional scaling (NMDS) multivariate analysis. We further tested whether photosynthetic microbial diversity, richness, abundances, chlorophyll-*a* content, ETR, and C fixation rates differed between bryosphere types using linear mixed-effects models taking into account the unbalanced design (12 plots in peatland and 25 plots in the rainforest; ANOVA with Type III sum of squares). Every linear mixed-effects model used ecosystem type (i.e., tropical rainforest or peatland) as a fixed effect and bryophyte species nested into ecosystem type as a random factor on the intercept to take into account potential bias related to bryophyte taxonomy found in each plot. Finally, we identified the drivers of photosynthetic microbial C fixation rates by means of structural equation modelling (SEM, Fig. S1; Table S2). Further details on statistical analyses are given in supplementary methods. All statistical analyses were performed using R version 4.0.2 [46].

## Results

### Diversity and composition of photosynthetic microbial communities

**Fig. 1:**
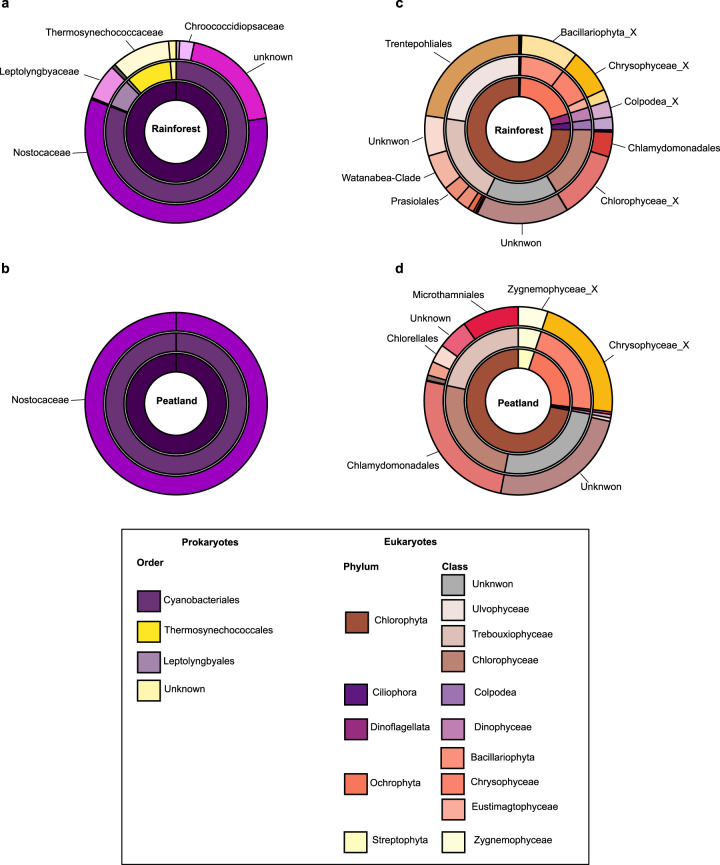
Relative abundance and proportions of dominant photosynthetic bacteria and protists in the boreal peatland and tropical rainforest. Donut plots showing the relative abundance of the different photosynthetic taxa identified with 16 S (**a**, **b**) and 18 S (**c**, **d**) metabarcoding and averaged for each climatic area. The inner, middle and external circles show the relative abundance at the phylum, class and order levels, respectively.

**Fig. 2:**
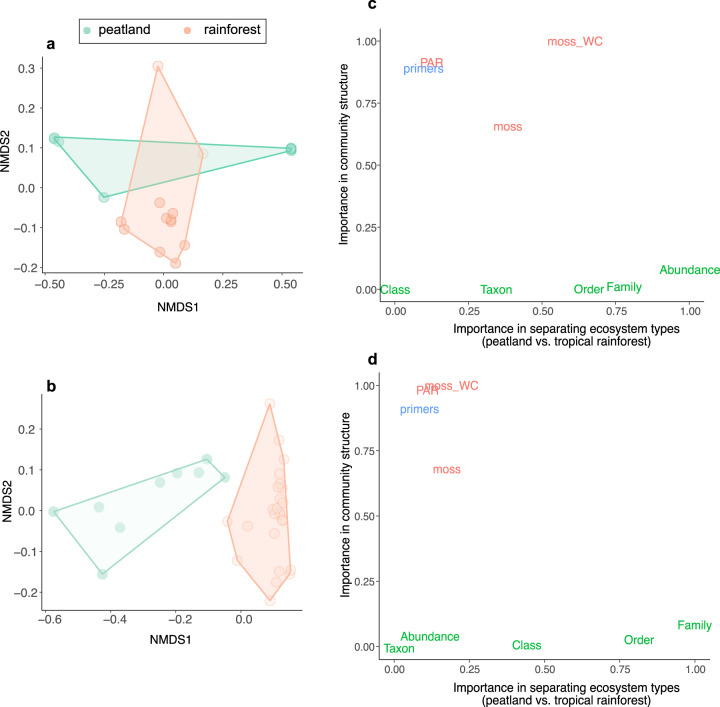
Divergence of photosynthetic bacterial and protists community structure between the boreal peatland and the tropical rainforest and their drivers. NMDS of the communities of photosynthetic bacteria and protists based on 16 S and 18 S gene amplicon sequencing data, respectively (**a**, **b**). Variables of importance from random forests models used to classify each type bryosphere based on the relative abundance of taxa (in green), environmental (in red) and technical variables (in blue) under unsupervised (x-axis) and supervised (y axis) mode, and including primers (reward and forward), bryophyte identity, bryophyte water content (%) and light availability ﻿(**c**, **d**). All values are variable importance from Random Forest models (normalized Gini index, see Methods); points that are further to the right on the x axis have more importance in separating studies, whereas points that are higher up on the y axis have more importance in community structure.

We obtained a total of 16,807 curated 16 S photosynthetic reads and 22,080 curated 18 S photosynthetic reads from the rainforest bryophyte samples, while 5911 curated 16 S photosynthetic reads and 1498 curated 18 S photosynthetic reads were found in the peatland bryophyte samples, respectively. The relative abundance of photosynthetic reads varied strongly across climatic regions. Photosynthetic bacteria represented on average 7.7% and 3.5% of the total 16 S reads in the peatland and the rainforest, respectively. Photosynthetic protists constituted a large part of the total 18 S reads in peatlands with 21%, but only 4% in the rainforest. OTUs clustering (97%) sequence similarity resulted in 9280 non-singleton prokaryotic OTUs (rainforest = 8231, peatland = 1049) and 4534 non-singleton eukaryotic OTUs (rainforest = 3788, peatland = 746), respectively. Following rarefaction at the sample level, we found a total of 165 OTUs for photosynthetic bacteria and 499 OTUs for photosynthetic protists. OTUs richness was by far the highest in rainforest with on average 22 photosynthetic bacteria and only 2 photosynthetic bacteria in peatland (χ^2^_(1, 1)_ = 20.4, *P* < 0.001), while photosynthetic protists’ richness was comparable between the two climatic regions (rainforest = 16; peatland = 13; (χ^2^_(1, 1)_ = 2.4, *P* = 0.12). Photosynthetic diversity (Shannon’s diversity index) showed similar patterns than richness for photosynthetic bacteria (rainforest = 11.7, peatland = 1.4; χ^2^_(1, 1)_ = 10.6, *P* < 0.001) and protists (rainforest = 6.9, peatland = 6.8; χ^2^_(1, 1)_ = 0.02, *P* = 0.88). Furthermore, we found important discrepancies in the composition and the structure of photosynthetic microbial communities among climatic regions (photosynthetic bacteria: *R²* = 0.28, *F*_(1, 17)_ = 6.8, *P* < 0.001; photosynthetic protists: *R²* = 0.28, *F*_(1, 35)_ = 12.2, *P* < 0.001). About 80% of prokaryotic OTUs and >50% of eukaryotic OTUs, respectively, were specific to a climatic region, resulting in contrasted communities composition (Fig. 2a, b). Photosynthetic bacteria in rainforest were dominated by Nostocaceae, Leptolyngbyaceae and Thermosynechococcaceae, while only Nostocaceae were found in peatland (Fig. 1a, b). Similarly, photosynthetic protists were more diverse in the rainforest and dominated by Bacillariophyta, Chrysophyceae, Chlorophyceae, mixotrophic Colpodea, and Trentepohliales while Zygnemophyceae, Chrysophyceae, and Chlamydomonadales were the main representative class in peatland (Fig. 1c, d).

These patterns of photosynthetic diversity and community composition could be plagued with potential technical biases related to the merging of different data sets. We thus quantified the importance of both environmental and technical factors alongside taxa abundance and occurrence in differentiating communities’ structures using random forests models (Fig. 2c, d). Both environmental and technical factors were poorly informative in separating data sets; the Family and Order taxonomic levels were the most important variables in both photosynthetic bacteria and protists datasets. By contrast, environmental factors such as bryophyte WC and light intensity were highly important for structuring the photosynthetic communities, followed by technical factors (forward and reverse primers; Fig. 2c, d). This indicates that the observed differences among photosynthetic communities’ structures are somehow confounded with technical factors and that our results about diversity and richness have to be taken with care.

### Photosynthetic microbial abundance and metabolic performance

**Fig. 3:**
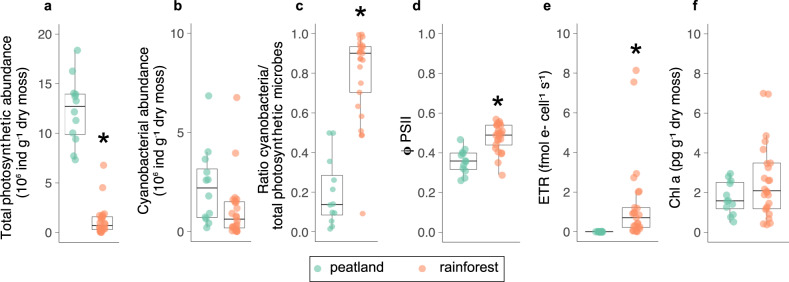
Photosynthetic microbial properties. The abundance of all photosynthetic microbes (**a**), cyanobacteria (**b**), and the ratio between cyanobacterial abundance and total photosynthetic microbial abundance (**c**) in each type of bryosphere. Photosynthetic efficiency (ΦPSII, **d**), photosynthetic electron transport rate per cell (ETR) (**e**), and chlorophyll-a cellular content of photosynthetic microbes in both types of bryosphere (**f**). Significant differences (*P* < 0.05, ANOVA Type III error) are indicated by asterisks.

Total microbial photosynthetic abundance was 5 times higher in the peatland (12.3 ± 3 × 10^6^ cells g^−1^ dry bryophyte) than in the rainforest (2.4 ± 6 × 10^6^ cells g^−1^ dry bryophyte; χ^2^_(1, 1)_ = 14.4, *P* < 0.001; Fig. 3a). Cyanobacteria represented on average 19% and 95% of the total photosynthetic abundances in the peatland and the rainforest, respectively, showing strong differences among ecosystem types (Fig. 3b, c). In terms of photosynthetic efficiency, we found that the quantum yield of photosystem II (ΦPSII) remained high in both climatic regions, although it was higher in the rainforest (averaged ΦPSII = 0.48) than in the peatland (averaged ΦPSII = 0.36; χ^2^_(1, 1)_ = 25.9, *P* < 0.001, Fig. 3d). Photosynthetic electron transport rates (ETR) and Chl*a* contents showed similar patterns than ΦPSII with higher values in the rainforest than in the peatland (ETR: χ^2^_(1, 1)_ = 12.2, *P* < 0.001; Chla: χ^2^_(1, 1)_ = 2.1, *P* = 0.15; Fig. 3e, f).

### Microbial contribution to the bryosphere C fixation rate

**Fig. 4:**
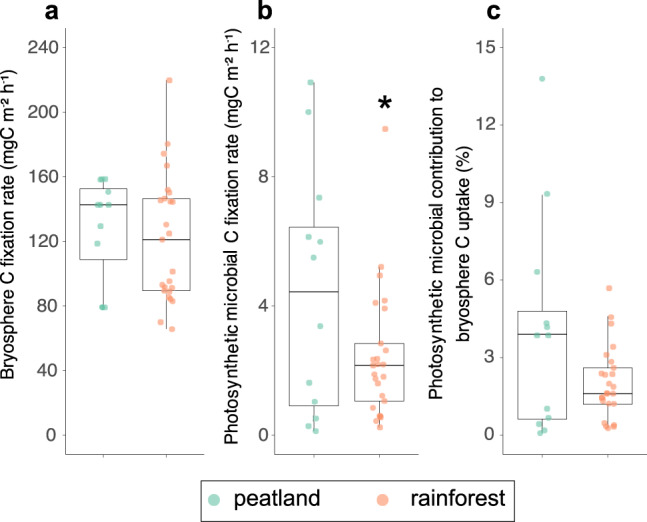
Photosynthetic microbial contribution to bryophyte C uptake. Microbial C fixation (**a**), bryosphere C fixation (**b**) and contribution of photoautotrophs to bryosphere C fixation (**c**) in both types of bryosphere. Significant differences (*P* < 0.05, ANOVA type III) are indicated by asterisks.

On average, the total C fixation rate of the bryosphere was similar in both climatic regions with rates of ~128 (min – max: 79–158) mg CO_2_.h^−1^.m^−2^ in the peatland and ~122 (min – max: 65–220) mg CO_2_.h^−1^.m^−2^ in the rainforest (χ^2^_(1, 1)_ = 0.46, *P* = 0.49, Fig. 4a). At the photosynthetic microbial level, we found different C fixation rates between the peatland and the rainforest (χ^2^_(1, 1)_ = 5.9, *P* = 0.015, Fig. 4b). Photosynthetic microbes fixed on average 4.4 (min – max: 0.12–10.9) mg CO_2_.h^−1^.m^−2^ in the peatland and 2.4 (min – max: 0.24 –9.5) mg CO_2_.h^−1^.m^−2^ in the rainforest. These microbial C rates translated into an averaged contribution of 4.1% (min – max: 0.1% − 13.8%) and 2.0% (min – max: 0.3% − 5.7%) to total bryosphere C fixation rate in the peatland and the rainforest, respectively (Fig. 4c), although these differences were not significant (χ^2^_(1, 1)_ = 0.31, *P* = 0.58).

**Fig. 5:**
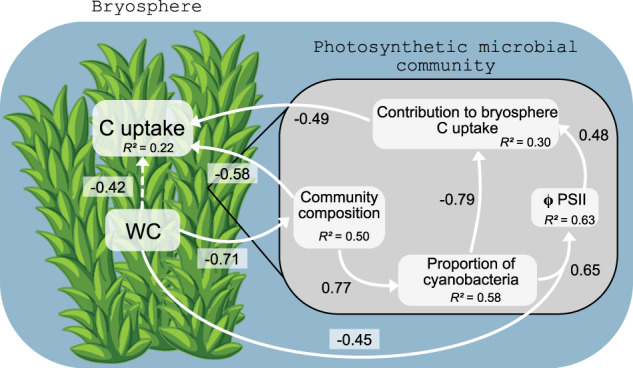
Structural equation model reflecting the main drivers of the contribution of photosynthetic microbes to bryosphere C uptake. Only standardized path coefficients with *P* < 0.05 are shown. Ratio C/P: ratio between the abundance of cyanobacteria and total abundance of photosynthetic microbes. The amount of variance explained (*R*^*2*^) for each response variable are given in their respective box. The global fit of the model was very good: AIC = 48.9, Fisher’s C = 6.86, *P* = 0.51. Dashed lines are nearly significant linkages (0.05 < *P* < 0.1).

Structural equation modelling (SEM) allowed us to identify the main drivers of the contribution of photosynthetic microbes to the bryophyte C uptake (Fig. 5). First, it showed that local conditions (bryophyte water content and light availability) directly determined photosynthetic microbial community composition (r∂ = −0.71) and its photosynthetic efficiency (ΦPSII; r∂ = −0.45). Second, the model revealed that the occurrence of photosynthetic protists was a pivotal factor in determining the contribution of photosynthetic microbes to the bryophyte C uptake. Specifically, the occurrence of photosynthetic protists strongly determined the ratio cyanobacteria:total photosynthetic microbial abundance in the community (r∂ = 0.77), which in turn determined the microbial photosynthetic efficiency (ΦPSII, r∂ = 0.65) and microbial CO_2_ fixation rates (r∂ = −0.79). In other words, photosynthetic microbial communities with more cyanobacteria than photosynthetic protists fixed less C and therefore contributed less to the total C uptake of the bryophyte. Finally, our results highlighted that total bryosphere C fixation directly depended on photosynthetic community composition (r∂ = −0.58).

## Discussion

﻿Our study provides the first attempt to investigate the changes in bryophyte-associated photosynthetic microbial communities across contrasting ecosystems. Our investigations clearly show that photosynthetic microbes associated with bryophytes have the capacity to take up atmospheric C, and therefore potentially affect rates of ecosystem-level net C exchanges with the atmosphere. Even though we focus on different types of bryosphere at a single date, our results are consistent with previous cryptogamic estimations [14]. More particularly, we show that photosynthetic microbes fix more C in the boreal peatland than in the tropical rainforest. In-site conditions such as light availability and bryophyte WC were important determinants of this pattern, as well as the abundance of photosynthetic protists in the microbiome. ﻿Our work provides new insights into the ecology and biogeography of terrestrial photosynthetic microbes and advances our understanding of the potential vulnerabilities of photosynthetic microorganisms’ diversity and C fixation rates to changing environmental conditions.

### Photosynthetic microbial communities’ composition varies with the bryospheres properties

Bryophytes are known to harbour very specific microbial communities across species [31, 47–49] and/or environmental gradients [39, 50]. For example, moss-associated bacterial communities from northern ecosystems are known to be structured by host identity and phylogeny [31, 49, 51]. Our study extends these previous findings by encompassing photosynthetic bacteria and protists sampled across two distinct climatic conditions. Particularly, we show that bryophyte-associated photosynthetic communities strongly diverge between boreal and tropical regions, which corroborates previous findings in mosses and soils on protists [2, 23, 50]. Photosynthetic bacteria were by far more diverse and relatively abundant in rainforest than in peatland, showing opposite patterns from most recent global studies in soils [20]. The richness and diversity of photosynthetic protists were comparable between the rainforest and the peatland, which differs from most recent observations in moss-associated microbiomes [50] and phytoplankton [52] but not from soils [23].

Our findings revealed that Nostocaceae was the most important class of photosynthetic bacteria in both ecosystem types, which is consistent with previous findings on moss-associated bacterial communities from northern ecosystems [51]. It however differs from soils where Oscillatoriales are usually the most represented class [20]. Similarly, our results evidenced that the most important classes of photosynthetic protists in both types of bryosphere, (i.e., Chlorophyceae, Chrysophyceae, Trebouxiophyceae, Eustimagtophyceae and Bacillariophyta) slightly diverged from soils usually dominated by Chlorophyceae, Trebouxiophyceae and Chrysophyceae [2]. These findings suggest that distinct assembly processes govern photosynthetic bacterial and protist communities composition between moss and soil [53]. For example, bryophytes may create specific micro-habitats which select for a distinct adapted community compared to soils. Bryophyte inherent factors such as moisture content [19], pH [54], nutrients availability [55] or even host genotypic clines [51, 56] may all contribute to photosynthetic community dissimilarity between bryophytes and soils. Other factors such as physiochemical plant traits may also play a role in shaping bryophyte-associated photosynthetic microbiomes [48, 57]. Bryophytes actively produce and excrete bioactive metabolites to their surroundings, such as polyphenols [58–60], flavonoids [61], carbohydrates [61, 62] and tannins [61, 63]. These compounds have been related to compositional differences in moss-associated microbial communities [39, 57]. Many of these metabolites possess antimicrobial properties [64], and therefore could regulate microbial dispersal between bryophytes and soils in ecosystems.

### Drivers of photosynthetic microbial C uptake in the bryosphere

On average, we found 5.7 ± 1.2 × 10^6^ photosynthetic microbial cells per gram of dry bryophyte, which is consistent with previous findings in soils where photosynthetic microbes typically range between 10^4^ and 10^8^ cells per gram of dry soil [15]. According to our hypothesis, we found large differences in terms of photosynthetic microbial abundance among climatic zones, with by far, more abundant photosynthetic microbes in the boreal peatland than in the tropical rainforest. This pattern is consistent with the most recent estimations of fungal and bacterial density patterns in topsoil [65] and may result from several interconnected evolutionary and eco-physiological processes [66]. Environmental filtering most probably determined the survival and reproduction of specific photosynthetic microbes among climatic zones. In particular, our analysis revealed that bryophyte WC and light intensity were important determinants of photosynthetic protists distribution, and most probably their survival. Indeed, the morphological features of *Sphagnum* bryophytes allow them to store more water than any other bryophyte genus [67]. On the opposite, bryophytes on tree trunks are more prompt to water stress because the water does not stagnate. The constant repetition of wet-dry cycles on tree trunks according to precipitation patterns may limit the survival of photosynthetic protists who are more sensitive to drought than cyanobacteria [36, 68]. Furthermore, the light intensity may have limited photosynthetic protists’ growth in the rainforest. Although many photosynthetic phyla show optimal photosynthesis at low light intensity by optimizing light harvesting at low light flux [69, 70], cyanobacteria possess unique and highly-adaptable eco-physiological traits allowing them to capture light at very low intensities and a range of wavelengths that photosynthetic protists do not possess [71]. As a corollary, our results suggest that local conditions in the rainforest do not provide a suitable environment for photosynthetic protists’ survival rather than specifically promoting cyanobacterial growth.

### Photosynthetic microbiomes fix a significant amount of CO_2_ in the bryosphere

We show that bryophytes host unique communities of photosynthetic microbes, which partly explained divergences in CO_2_ assimilation rates observed across climatic zones. Photosynthetic microbes fixed on average 4.4 (0.1–10.9) mg CO_2_ h^−1^ m^−2^ in the peatland and 2.5 (0.24–9.5) mg CO_2_ h^−1^ m^−2^ in the rainforest, which represented about 4% (0.1–13.8%) and 2% (0.3–5.7%) of the total bryosphere C fixation, respectively. We acknowledge that our fluorescence-based measurements of microbial photosynthesis may have biased our estimates [72]. More particularly, the high proportion of cyanobacteria in the tropical rainforest may have underestimated their photosynthetic rates, as chlorophyll fluorescence measurements are often underestimated in communities dominated by cyanobacteria because of their divergent pigment packaging [73]. Nevertheless, microbial photosynthetic C fixation rates from bryophytes estimated using fluorescence have been shown to be of similar magnitude as C fixation rates quantified using a gas analyzer [9], providing confidence to our estimates. Furthermore, our results are in line with recent estimations [9], where a contribution of ~10% has been found in temperate, subarctic, and arctic peatlands. Our results further suggest that the sensitivity of photosynthetic protists to poor light intensity and low water content conditions explained the lower microbial photosynthetic C fixation rates in the tropical rainforest compared to the boreal peatland (Fig. 5). This is despite higher photosynthetic efficiency per photosynthetic cell (ΦPSII and ETR) in the rainforest than in peatland (Fig. 3). Cyanobacteria are known to perform better at high temperatures [74]. They grow and replicate faster under temperatures approaching their physiological optima—ranging between 20 °C and 40 °C [71, 75]—and have a net advantage over photosynthetic protists when temperatures increase above 20 °C [71], at least under moist conditions [74]. Repeated wet-dry cycles in the rainforest bryosphere most probably cancelled the benefits of high temperatures on cyanobacterial growth, which suggest that the temperature dependence of cyanobacteria in the rainforest tends to be controlled by bryophyte’s moisture content.

## Conclusion

In conclusion, our study presents a comprehensive assessment of the composition and function of photosynthetic microbes in bryophytes from different biomes. Our results show that photosynthetic microbes from different bryospheres are capable of atmospheric CO_2_ fixation under a wide range of environmental and local conditions. However, significant changes in the magnitude of this C fixation rate should be expected as bryophyte moisture content changes. Terrestrial systems globally take up ~120 Gt of C each year [76], representing an important ecosystem service mitigating climate change. Net C exchange between the atmosphere and land is a delicate balance driven by eco-physiological processes, and a shift in any of these can impact terrestrial C cycling. Our results demonstrate that photosynthetic microbes are important players in ecosystem C uptake and suggest they effectively support bryosphere C fixation. More generally, our work demonstrates the utility of studying plant microbiomes for understanding plant survival and broadens our understanding of how host-microbe interactions contribute to C dynamics in northern and tropical ecosystems.

## Supplementary information

Supplementary information

## Data Availability

Data and codes related to this paper are available from Figshare (10.6084/m9.figshare.20170535).
