# Supplementary material for: Photosynthetic microorganisms effectively contribute to bryophyte CO2 fixation in boreal and tropical regions
Source: ISME Commun. 2022 Jul 28;2:64. doi: 10.1038/s43705-022-00149-w (PMC9723567; doi:10.1038/s43705-022-00149-w)
Supplement: Supplementary file 1 — Supplementary information [file 43705_2022_149_MOESM1_ESM.pdf]

# Supplementary Information for

# Photosynthetic microorganisms effectively contribute to bryophyte $CO_2$ fixation in boreal and tropical regions

Vincent E.J. Jassey, Samuel Hamard, Cécile Lepère, Régis Céréghino, Bruno Corbara, Martin Küttim, Joséphine Leflaive, Céline Leroy, and Jean-François Carrias

Content:

Supplementary Methods
Supplementary Tables : 2
Supplementary Figures : 3
Supplementary references

## Supplementary methods

*DNA extraction, amplification, and sequencing*

To enhance the recovery of photosynthetic microbes from a relatively large amount of bryophyte material, we separated microbes from bryophytes following a protocol adapted from [1]. In the field, approximately 3 g of previously homogenized bryophyte material was collected in each plot and transferred into a 50- ml tube with 25 ml of distilled water and gently shaken intermittently for 1 min by hand. Then, the solution was passed through a 150 µm mesh to remove bryophyte material, and filtered through a 0.8 *µ*m pore-size Supor-membrane (Pall, Ann Arbor, MI). Finally, the filter was frozen until further analyses. DNA was extracted using the DNeasy PowerSoil Pro Kit (Qiagen) following the manufacturer's instructions with an additional step of mechanical grinding with Bead Beater MM301 RETSCH for the rainforest samples and using a FastPrep-96™ for the peatland samples (MP biomedicals). DNA was quantified using a Nanodrop ND-1000 spectrophotometer and a Qubit with the DNA HS Assay kit (Invitrogen). Extracts were stored at -20°C before proceeding to DNA amplification. Amplification of the 16S rRNA and 18S rRNA genes was performed using universal primers for both types of samples (Table S1). We acknowledge that the sets of primers slightly differ between peatland and rainforest samples. However, they were all generalist and both showed a high percentage of sequence matching, especially for the photosynthetic interested microbial groups (comparisons can be made on https://app.pr2-primers.org/) [2]. For the rainforest extracts, each PCR was performed in a total volume of 30 µL containing 6 µL of 5x Green GoTaq Flexi buffer, 2.4 µL of 25 mM $MgCl_2$, 0.18 µL of GoTaqG2 Flexi DNA Polymerase (Promega, 5 U/µL), 0.6 µL of 10 mM each dNTP, 0.3 µL of 50 mg/mL BSA and 0.6 µL of each 10 µM primer. For the peatland extracts, PCRs were conducted in a total volume of 50 µl containing 13 µl of Mastermix AmpliTaq Gold (Thermofisher), 1µl of each primer (0.4 µM final concentration), 35 ng of DNA (up to 10 µl) and completed to 50 µl with DNA-free water. PCR programs are presented in Table S1. The DNA sequencing was performed by the GeT-PlaGe platform (Genotoul, Toulouse, France) with Illumina MiSeq technology and using the V3 chemistry (2x250 bp).

Paired-end fastq sequences were analysed using the FROGS pipeline (Find Rapidly Operational Taxonomic Units Galaxy Solution) on the Galaxy platform [3]. Paired-end reads were merged using Vsearch (10% of mismatch; [4]). Sequences were filtered based on their length and primer mismatches were removed. Sequences were then dereplicated and

clustered into OTUs using the Swarm clustering method with an aggregation distance of 3 [5]. Chimera were identified and removed using Vsearch. Filters were then applied to remove singletons. OTUs were assigned at different taxonomic levels using Blast. We used the Silva 138 database [6] for the 16S assignation and the PR2 v4.12.0 database [7] for the 18S assignation. After these steps, the 25 rainforest samples contained 453,791 (16S) and 514,768 (18S) curated reads, belonging to 9,506 (16S) and 4,373 (18S) OTUs. The 12 peatland samples contained 258,123 (16S) and 258,123 (18S) curated reads, belonging to 1,409 (16S) and 746 (18S) OTUs.

Further bioinformatics analyses were conducted in R [8] with the Phyloseq R package [9]. Photosynthetic bacteria and protists sequences obtained with 16S and 18S markers were retrieved by removing chloroplast and mitochondria and bryophyte-associated sequences. Among the microbial OTUs obtained, we further identified the photosynthetic ones at the genus level. We considered that an OTU was photosynthetic if its related genus was referred to as strictly photosynthetic or mixotrophic. We included endosymbiotic mixotrophs [10] such as mixotrophic ciliates and testate amoebae among the photosynthetic OTUs of the 18S marker. For the 25 rainforest samples, we obtained 16,807 (16S) and 22,080 (18S) photosynthetic reads, belonging to 144 photosynthetic bacteria OTUs and 301 photosynthetic eukaryotic OTUs, respectively. For the 12 peatland samples, we obtained 5,911 (16S) and 1,498 (18S) photosynthetic microbial sequences, belonging to 9 photosynthetic bacteria OTUs and 198 photosynthetic protists OTUs, respectively. Because our number of photosynthetic microbial reads was correlated with our number of photosynthetic microbial OTUs, we performed a rarefaction for each sample to alleviate sequencing artefact. For the 25 rainforest samples, we obtained 2,160 photosynthetic microbial 16S sequences and 1,850 18S sequences, belonging to 100 and 188 photosynthetic microbial OTUs, respectively (Supplementary Figure S2a). For the 12 peatland samples, we obtained 252 photosynthetic 16S sequences and 666 18S photosynthetic sequences, belonging to 6 and 80 photosynthetic microbial OTUs, respectively (Supplementary Figure S2b). Finally, we checked the profiles of the rarefaction curves; not every sample reached saturation. We, therefore, kept only the samples near saturation using the same cutting threshold for both rainforest and peatland samples. We respectively lost 13 and 0 samples for 16S and 18S reads in the rainforest, and 5 and 3 samples in peatland. Richness, diversity, and community composition analyses were performed on these remaining samples only.

## Statistical analyses

*Merging raw sequence data and validation*. The two raw sequence data sets in the rainforest and the peatland, respectively, were initially conducted to stand as separate studies, and as such, show some methodological divergences. In particular, the primer pairs used to produce amplicons slightly differ among the two studies (Table S1), which could be more important in explaining differences between rainforest and peatland photosynthetic microbial communities than abiotic and biotic drivers of photosynthetic microbial community structure [11]. To test such an assumption, we used a random forests approach that took methodological and technical biases into account in addition to abiotic variables and the importance of taxa in explaining the structure of photosynthetic microbial communities in both climatic regions.

Following [11], we ran random forests models into supervised and unsupervised modes with the presence/absence and relative abundance of taxa as well as bryophyte identity, bryophyte WC, light intensity, primers pairs, and photosynthetic microbial taxonomy (Class, Order, Family,…) as explanatory variables. When run in unsupervised mode, the importance of explanatory variables describes how effective they are at separating the observed data from randomized synthetic data, while supervised mode shows the importance of explanatory variables to explain how effective they are at separating photosynthetic microbial communities from both climatic regions (response variable) [11]. In both modes, we quantified the importance of explanatory variables using the Gini index. Several parameters can be adjusted in random forests models but most of them remained set at their default value (e.g. mtry, which is the number of variables randomly sampled by the model for a split in the constituent tree). We, however, used a high number of trees (ntree = 2000) for each forest to achieve the stable importance of explanatory variables.

*Drivers of photosynthetic microbial C fixation rates*. We used structural equation modelling (SEM; [12]) to study the complex interactions determining the contribution of photosynthetic microbes to bryophyte C uptake. Following current knowledge on photosynthetic microbial eco-physiology [13, 14], we developed an a priori model of hypothesized relationships (Fig. S2, Table S2) within a path diagram allowing a causal interpretation of microbial contribution to bryophyte C fixation in relation with the microbial C fixation rates, photosynthetic microbial community structure (NMDS site scores from 16S and 18S data sets), metabolic parameters (e.g. photosynthetic efficiency), the abundance of cyanobacteria and total photosynthetic

microbial abundance, as well as environmental parameters (bryophyte water content, PAR, and bryophyte identity; Table S2). All components of the model were united and tested using the *psem* function from the piecewiseSEM R package [15]. The goodness-of-fit of our a priori SEM model was evaluated using Akaike Information Criterion (AIC) and Fisher's statistic. Based on the outputs of the a priori model and by step-wise exclusion/selection of variables, we found the model minimizes the AIC. The main drivers of microbial C fixation were determined from this final model.

All statistical analyses were performed using R version 4.0.2 [8].

**Table S1: Primers and PCR programs used for the meta-barcoding markers used in the study. Percentage of sequence matching has been taken from the application developed in Vaulot et al. (2021).**

| | Analyses | 16S | % sequence matching | 18S | % sequence matching |
|---|---|---|---|---|---|
| Peatland | Primers | 515F<br>928R | | TAReuk454FWD1<br>TAReukREV3 | |
| | Sequence of the primers (5' – 3') | 515F : GTGYCAGCMGCCGCGGTA | 96.4 | TAReuk454FWD1:CCAGCASCYGCGGTAATTCC | 92.8 |
| | | 928R: GGYGTCATATTYGGTGG | 91.8 | TAReukREV3: ACTTTCGTTCTTGATYRA | 71.5 |
| | Program of the PCRs | Activation : 10 min – 95°C<br><br>30 cycles:<br>60 s – 94°C<br>40 s – 65°C<br>30S – 72°C<br><br>Final elongation : 10 min – 72°C | | Activation : 10 min – 95°C<br><br>45 cycles:<br>30 s – 94°C<br>45 s – 47°C<br>60S – 72°C<br><br>Final elongation : 10 min – 72°C | |
| | Remarks | NA | | 45 cycles needed to obtain even amplification yields. We followed the method of Seppey et al. (2020) | |
| Rainforest | Primers | 515 Foward<br>909 Reverse | | 515 Foward<br>951 Reverse | |
| | Sequence of the primers (5' – 3') | 515F : GTGYCAGCMGCCGCGGTA | 96.4 | 515F : GTGYCAGCMGCCGCGGTA | 93.0 |
| | | 909R : CCCCGYCAATTCMTTTRAGT | 91.8 | 951R : TTGGYRAATGCTTTCGC | 75.3 |
| | Program of the PCRs | Activation : 10 min – 94°C<br><br>35 cycles:<br>60 s – 94°C<br>60 s – 58°C<br>90S – 72°C<br><br>Final elongation : 10 min – 72°C | | Activation : 10 min – 94°C<br><br>35 cycles:<br>60 s – 94°C<br>60 s – 55°C<br>90S – 72°C<br><br>Final elongation : 10 min – 72°C | |

**Table S2: Components of hypotheses represented by the *a priori* structural equation model (Fig. S2).**

| Path | Causal hypothesis |
|---|---|
| 1 | Bryosphere's local conditions such as water content, light intensity and taxonomy select for certain photosynthetic microbial taxa. |
| 2 | Photosynthetic microbial community composition determines the photosynthetic efficiency of the community. |
| 3 | Photosynthetic microbial density varies according to the community composition of photosynthetic microbes. |
| 4 | The photosynthetic efficiency of photosynthetic microbes determines their growth and hence their total density. |
| 5 | Photosynthetic microbial efficiency strongly determines their C fixation rates. |
| 6 | Photosynthetic microbial density influence their C fixation rates. |
| 7 | Photosynthetic microbial C fixation significantly contribute to total bryosphere C fixation rate. |
| 8 | Photosynthetic microbial C fixation significantly supports bryosphere C uptake |

**Fig. S1**

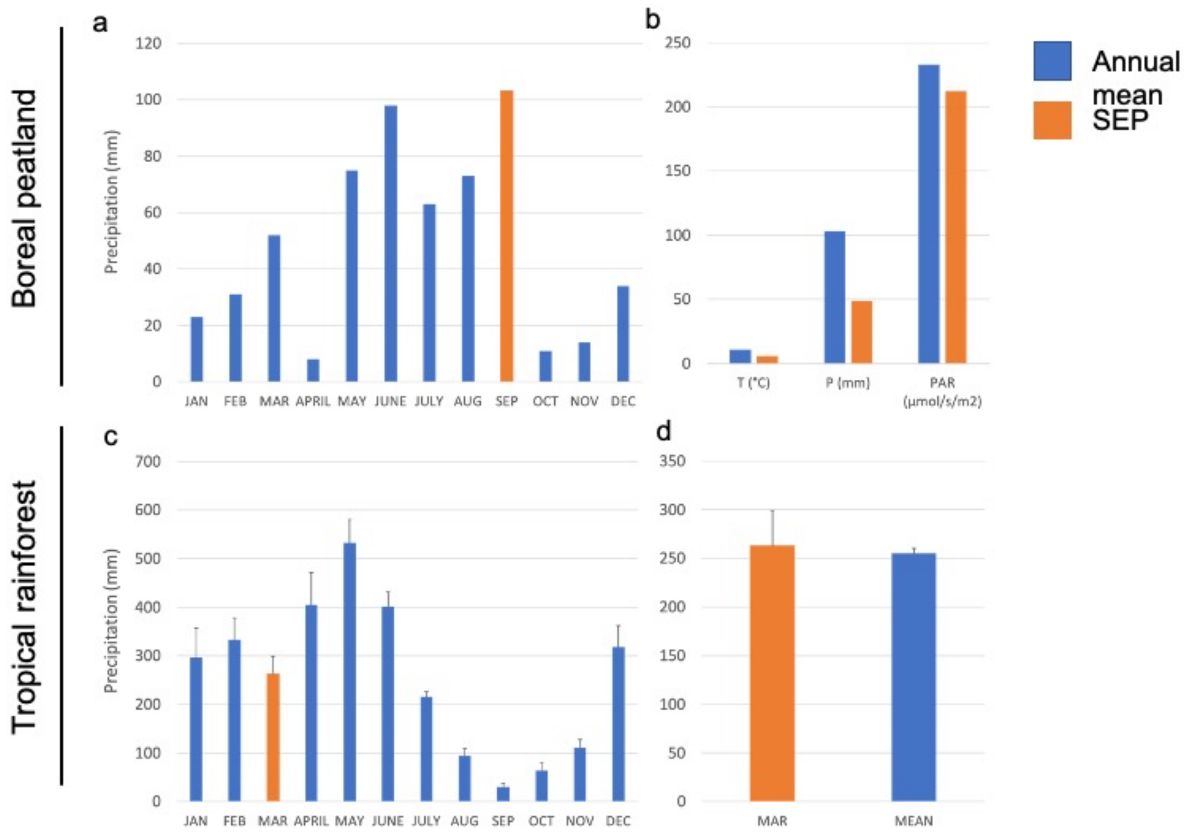


Summary of the microclimatic conditions in each site. Monthly precipitation in the boreal peatland (2019 data) and the tropical rainforest (average over 2009-2019 data). The bars in orange represent the conditions a month before sampling. (b) Microclimatic conditions in the boreal forest a month before sampling compared to mean annual values for temperature, precipitation, and light intensity (PAR). (c) Precipitation in the rainforest a month before sampling compared to mean annual precipitation. At both sites, these results show that we captured well the annual microclimatic conditions during our sampling campaigns.

**Fig. S2**

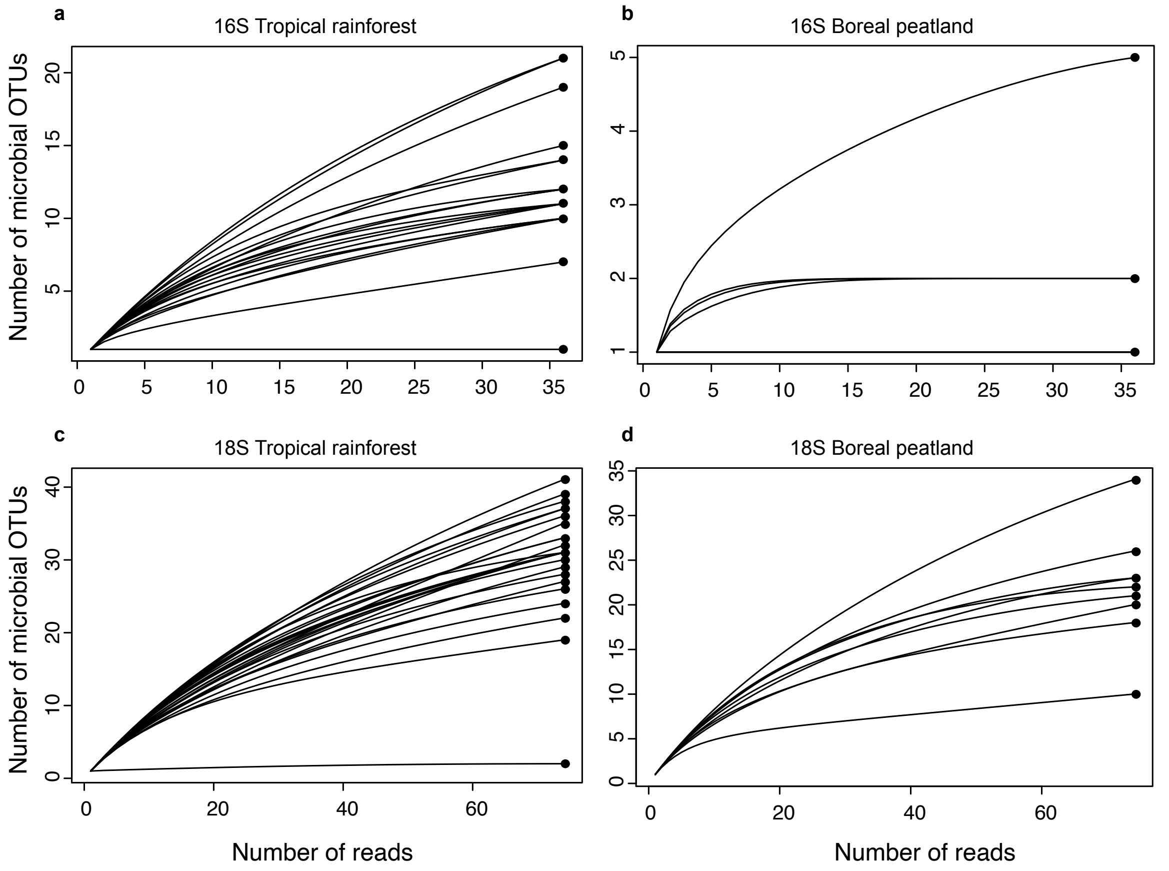


Rarefaction curves of photosynthetic microbial sequences after normalization of the number of sequences, in each sample 16S (a, b) and 18S (b, d) samples in the tropical rainforest and peatland.

**Fig. S3**

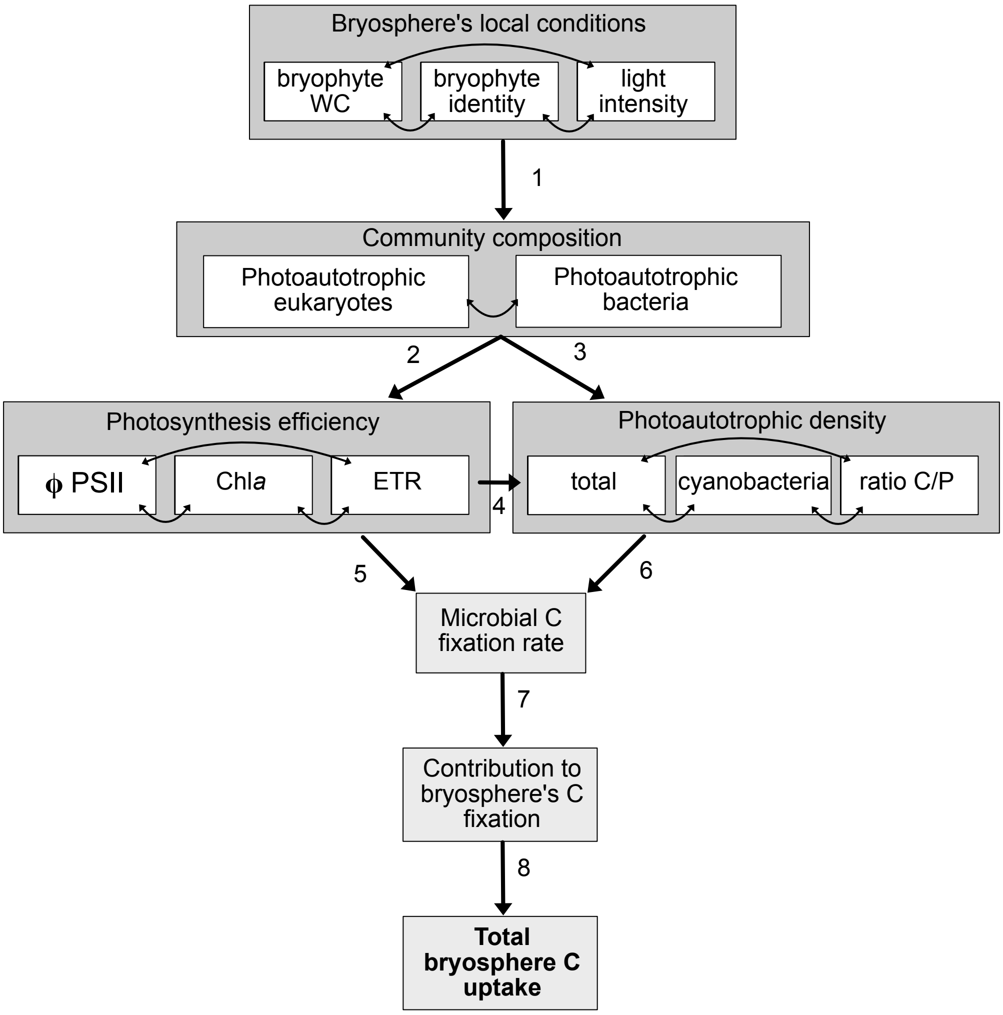


A priori conceptual structural equation model (SEM) depicting pathways by which the local conditions of the bryosphere may affect the composition and the abundance of photosynthetic microbial communities, and how, in return, they may affect microbial C fixation. Numbers correspond to hypothetical pathways (Table S2). Double headed arrows indicate strong collinearity among potential descriptors.

## Supplementary references


1. Heger TJ, Giesbrecht IJW, Gustavsen J, del Campo J, Kellogg CTE, Hoffman KM, et al. High-throughput environmental sequencing reveals high diversity of litter and moss associated protist communities along a gradient of drainage and tree productivity. *Environ Microbiol* 2018; **20**: 1185–1203.
2. Vaulot D, Geisen S, Mahé F, Bass D. pr2-primers: an 18S rRNA primer database for protists. *Mol Ecol Resour* 2021; 1755–0998.13465.
3. Escudié F, Auer L, Bernard M, Mariadassou M, Cauquil L, Vidal K, et al. FROGS: Find, Rapidly, OTUs with Galaxy Solution. *Bioinformatics* 2018; **34**: 1287–1294.
4. Rognes T, Flouri T, Nichols B, Quince C, Mahé F. VSEARCH: A versatile open source tool for metagenomics. *PeerJ* 2016; **2016**: e2584.
5. Mahé F, Rognes T, Quince C, de Vargas C, Dunthorn M. Swarm: robust and fast clustering method for amplicon-based studies. *PeerJ* 2014; **2**: e593.
6. Quast C, Pruesse E, Yilmaz P, Gerken J, Schweer T, Yarza P, et al. The SILVA ribosomal RNA gene database project: improved data processing and web-based tools. *Nucleic Acids Res* 2013; **41**: D590–D596.
7. Guillou L, Bachar D, Audic S, Bass D, Berney C, Bittner L, et al. The Protist Ribosomal Reference database (PR2): a catalog of unicellular eukaryote small sub-unit rRNA sequences with curated taxonomy. *Nucleic Acids Res* 2013; **41**: 597–604.
8. R Core Team. R: A language and environment for statistical computing. R Foundation for Statistical Computing, Vienna, Austria. 2019.
9. McMurdie PJ, Holmes S. phyloseq: An R Package for Reproducible Interactive Analysis and Graphics of Microbiome Census Data. *PLoS One* 2013; **8**: e61217.
10. Mitra A, Flynn KJ, Tillmann U, Raven JA, Caron D, Stoecker DK, et al. Defining Planktonic Protist Functional Groups on Mechanisms for Energy and Nutrient Acquisition: Incorporation of Diverse Mixotrophic Strategies. *Protist* 2016; **167**: 106–120.
11. Ramirez KS, Knight CG, de Hollander M, Brearley FQ, Constantinides B, Cotton A, et al. Detecting macroecological patterns in bacterial communities across independent studies of global soils. *Nat Microbiol* 2018; **3**: 189–196.
12. Grace JB, Adler PB, Harpole WS, Borer ET, Seabloom EW. Causal networks clarify productivity–richness interrelations, bivariate plots do not. *Funct Ecol* 2014; **28**: 787–798.
13. Huete-Ortega M, Cermeño P, Calvo-Díaz A, Marañón E. Isometric size-scaling of metabolic rate and the size abundance distribution of phytoplankton. *Proc R Soc B Biol Sci* 2012; **279**: 1815–1823.
14. Padfield D, Buckling A, Warfield R, Lowe C, Yvon-Durocher G. Linking phytoplankton community metabolism to the individual size distribution. *Ecol Lett* 2018; **21**: 1152–1161.
15. Lefcheck JS. piecewiseSEM: Piecewise structural equation modelling in r for ecology, evolution, and systematics. *Methods Ecol Evol* 2015.
